# Gut microbial community supplementation and reduction modulates African armyworm susceptibility to a baculovirus

**DOI:** 10.1093/femsec/fiac147

**Published:** 2022-12-06

**Authors:** Philip Donkersley, Annabel Rice, Robert I Graham, Kenneth Wilson

**Affiliations:** Lancaster Environment Centre, Lancaster University, Lancaster LA1 4YQ, United Kingdom; Lancaster Environment Centre, Lancaster University, Lancaster LA1 4YQ, United Kingdom; Department of Rural Land Use, SRUC, Craibstone Campus, Aberdeen AB21 9YA, United Kingdom; Lancaster Environment Centre, Lancaster University, Lancaster LA1 4YQ, United Kingdom

**Keywords:** African armyworm, Gut, microbiome, nucleopolyhedrovirus, symbiosis

## Abstract

Gut microbiota stimulates the immune system and inhibits pathogens, and thus, it is critical for disease prevention. Probiotics represent an effective alternative to antibiotics used for the therapy and prevention of bacterial diseases. Probiotic bacteria are commonly used in vertebrates, although their use in invertebrates is still rare. We manipulated the gut microbiome of the African Armyworm (*Spodoptera exempta* Walker) using antibiotics and field-collected frass, in an attempt to understand the interactions of the gut microbiome with the nucleopolyhedrovirus, SpexNPV. We found that *S. exempta* individuals with supplemented gut microbiome were significantly more resistant to SpexNPV, relative to those with a typical laboratory gut microbiome. Illumina MiSeq sequencing revealed the bacterial phyla in the *S. exempta* gut belonged to 28 different classes. Individuals with an increased abundance of *Lactobacillales* had a higher probability of surviving viral infection. In contrast, there was an increased abundance of *Enterobacteriales* and *Pseudomonadales* in individuals dying from viral infection, corresponding with decreased abundance of these two Orders in surviving caterpillars, suggesting a potential role for them in modulating the interaction between the host and its pathogen. These results have important implications for laboratory studies testing biopesticides.

## Teaser

Our study examines how lab strains of a widespread insect pest species (the African Armyworm) have a distinct gut microbiome and the role that gut microbes play in affecting resistance to nucleopolyhedrovirus, SpexNPV. Through a combination of antiobiotic control and field-collected frass innoculations, we demonstrate that inoculation of the field microbiome of this pest species into lab strains increases resistance to the virus, and that this is subsequently reversed when treated with a broad spectrum antibiotic.

## Introduction

As we work to introduce more ecological principles into immunology, natural phenomena such as multiple infections, host fitness trade-offs, and interactions with microbial symbionts are shaping how we study the interactions among hosts, microbial symbionts, and pathogens within a ‘hologenome’ concept (Rosenberg and Zilber-Rosenberg 2018). Animal models can help us in our understanding of how a microbiome can impact susceptibility to pathogens.

Both plants and animals are colonized by symbiotic microbial organisms that have beneficial and fundamentally important impacts on host biology. These organisms potentially represent a hologenome containing 150 times the number of functional genes as its host (Gill *et al*. 2006, Qin *et al*. 2010). Microbes can regulate plant and animal development, immune function and metabolism; clearly the importance of these organisms suggests a key role in the evolutionary origin and diversification of animal clades (Bäckhed *et al*. 2005, Janson *et al*. 2008, Frago *et al*. 2012, Douglas 2014, Flórez *et al*. 2015, Sudakaran *et al*. 2017). These integral host–microbe relationships have led to a conceptualization of animals as ‘holobionts’ (Janson *et al*. 2008, Frago *et al*. 2012, Sudakaran *et al*. 2017), superorganism-like entities composed of the host plus its microbiome. Disruption of a microbial community can lead to increased disease susceptibility (Hamdi *et al*. 2011, Mattila *et al*. 2012, Maes *et al*. 2016), through the loss of defensive symbionts (Kaltenpoth and Engl 2014, Flórez et al. 2015) or the abandonment of exploitable microbial niches (Harris *et al*. 2009, Lawley *et al*. 2012, Cariveau *et al*. 2014).

The African armyworm, *Spodoptera exempta* and its baculovirus *Spodoptera exempta* nucleopolyhedrovirus (SpexNPV) offer a robust model system for studying the impact of the gut microbiome on pathogen susceptibility. *Spodoptera exempta* is a major crop pest of sub-Saharan Africa. It is highly migratory and over multiple generations during a single outbreak season can travel thousands of kilometres (Brown and Swaine 1965, Rose *et al*. 1995). SpexNPV infects larvae through the ingestion of viral occlusion bodies (OBs). When the OBs enter the midgut, their protein coat is dissolved and virions are released into the midgut (Graham*et al*. 2012, Grzywacz *et al*. 2014). Virus proliferation in secondary infections of fat bodies leads to tissue destruction, with host death occurring typically within 4–7 days (Brown and Swaine 1965, Tinsley 1979). As the mode of action for this virus is to infect through gut tissue, the host gut microbiome is hypothesised to have an important role in modulating this infection.

The effects microbial symbionts have on the ecology and evolution of invertebrate hosts is a deep and diverse field of study (Buchner 1965, Ratzka et al.^2012^, Eleftherianos *et al*.^2018^, Paniagua Voirol *et al*. 2018, Jing *et al*. 2020). To our knowledge, very few studies beyond those focusing on a narrow group of host organisms (Aphidae or *Apis/Bombus*) (Vorburger *et al*. 2010, Kaltenpoth and Engl 2014), those focusing on the role of a single microbial symbiont (*Wolbachia*) (Graham *et al*. 2012, Pimentel *et al*. 2021), or using purely bioinformatics approaches (Xu *et al*. 2014, 2019) have explored in detail the roles microbial symbionts have in a host–pathogen system (Oliver *et al*. 2003, Russell *et al*. 2013, Kaltenpoth and Engl 2014, Xu *et al*. 2014, Borges *et al*. 2021). Recent studies on the black soldier fly (*Hermetia illucens*) and cockroaches have examined how diet impacts the gut microbiome, and that this has downstream impacts on antimicrobial peptide generation—an important factor in pathogen resistance (Akbar *et al*. 2018, Vogel *et al*. 2018, Wynants *et al*. 2019). Although gut microbiomes are highly species-specific (Brucker and Bordenstein 2013), their widespread role in shaping host evolution in the invertebrates (Moran *et al*. 2019) emphasises this systems suitability as a model of host–pathogen microbiome dynamics.

The aim of this study was to examine how supplementation or destruction of host gut microbiome impacts host susceptibility to a virus. Specifically, we addressed the following questions: (i) Can we manipulate the insect gut microbiome composition through diet? (ii) Is susceptibility to viral infection related to the diversity of gut microflora? (iii) Are specific members of the gut microbiome responsible for a defensive symbiosis, or is it due to a complex microbial community?

## Materials and methods

### Insect culture

A colony of *Spodoptera exempta* were maintained on a semi‐synthetic wheatgerm‐based diet that included a broad spectrum antibiotic (streptomycin 1.1 mg g^−1^ diet) to reduce bacterial contamination of the diet (Reeson *et al*. 1998, Vilaplana *et al*. 2010) at a constant temperature of 27°C under a 12 h light/dark cycle. The *S. exempta* culture was initiated from pupae collected in South Africa in 2014, with a generation time of ∼28 days, this amounts to approximately 72 generations of rearing on a diet containing antibiotics. Genetic diversity in the ‘primary culture’ was maintained through a cross-breeding programme and associated stud-book of adult moths maintained by laboratory technical staff (Wilson *et al*.^2021^). From the primary culture 50 pupae, each with a distinct genetic heritage were selected to begin a new sub-culture maintained with a microbial-supplemented artificial diet (defined as the ‘*probiotic* line’). A further 50 genetically distinct pupae were selected to begin a sub-culture maintained according to the standard laboratory diet (hereafter defined as the ‘*lab* line’). Within these sub-cultures, 100 adult moths were paired at the end of each generation to maintain genetic diversity within each subculture.

The *probiotic* line was reared on the same semi-synthetic diet, but with the antibiotic removed, instead replaced with frass (40 mg g^−1^ diet) from field-collected *S. exempta* caterpillars fed on grass and maize leaves in Tanzania. Caterpillars were collected from infested fields, taken to the field-station, and fed fresh vegetation; fresh frass was then collected and immediately refrigerated until suspension for use in experiments. The frass was added to 200 ml 1x phosphate buffer solution (pH 7.4) and placed in a shaking incubator for 10 min before being added to the diet. This culture was maintained for two generations, after which we confirmed restoration of the gut microbiome through Illumina MiSeq sequencing of the 16 s rRNA bacterial gene (see below). A subsample of ‘wild’ frass was used in a sequencing run also using the 16 s rRNA gene. The *lab* and *probiotic* lines were synchronized according to egg-lay date and larval emergence date for viral bioassays (see below). Each generation of larvae used in bioassays used third instar (L3) larvae selected equally from across the genetically distinct lines within each sub-culture (Supplementary Materials A).

### SpexNPV bioassay

The *lab* and *probiotic* lines were assayed for their response to challenge by SpexNPV using a standardized bioassay method. Briefly: we produced 1 mm³ cubes of the wheatgerm-based semi-artificial diet, to which we added 1 µL of 20% sucrose solution by treatment group (Table S1). L3 stage larvae were fed diet cubes individually in 96-well microtitre plates for 24 h before being transferred to individual diet pots for the remainder of the bioassay. Controls for each group were treated with 1 µL of sterile sugar solution. The bioassay was performed using an equal mix of 40 genetically-distinct isolates of SpexNPV collected from 12 locations in Tanzania in 2008–2010 (Graham *et al*. 2012).

To provide an LD80 dosage (predicted to kill 80% of larvae), we used a dosage of 2.5*10³ OBs (viral occlusion bodies: OBs) for each individual. Following the initial virus exposure, handling deaths were discounted and viral/fungal/bacterial deaths were confirmed and counted over each 24 h period until day 8 (D8) after virus exposure. On D14, all remaining survivors were killed for microbial community analysis. Each bioassay was performed using two treatment groups: *lab* line (n = 480) and *probiotic* line (n = 480), with controls (i.e. sterile sugar solution as above) for each group (n = 96). Each bioassay was replicated three times.

Two supplementary control groups were also tested. To control for the potential toxic effects of antibiotics interacting with the viral infections, a replicate (n = 480) of the *lab* line fed on their semi-artificial diet without antibiotics was bioassayed with the same virus dosage. To control for potential genetic selection effects in the host, a further virus bioassay was performed on the *probiotic* line, wherein it was crossed back onto the *lab* line semi-artificial diet with supplementary dosages of antibiotics (n = 480). Finally, development time is impacted by the gut microbiome (Prado and Almeida 2009), and immune responses vary according to development stage. To control for this the viral bioassays used L3 instar larvae that develop simultaneously across the lab, probiotic and control groups, thus we had already selected for individuals developing at the same rate.

### Microbial community analysis

Larvae were surface cleansed using Triton-X, then total gut content (crop, midgut, and rectum) was removed. Microbial DNA was extracted from each of the 240 caterpillar gut samples across each of the treatment groups (Table 1) using the QIAamp DNA Microbiome Kit (Qiagen Ltd, Crawley, UK). DNA extractions were performed according to manufacturers’ specifications with an additional bead-beating step to eliminate selective bias towards gram-negative bacteria (Lim *et al*. 2018). Individuals that died from viral infection were collected on the fourth day after viral dosage, whereas individuals that survived were collected on day 8, as this was the only practical way to guarantee their description as a survivor.

**Table 1.** Illumina MiSeq sequencing sample organisation, including number of post-filter reads across all samples within each treatment type.

Treatment	Bioassay	Result	n	Successful amplification	Post-filter reads
**Probiotic**	LD80	Survivors	48	37	2129617
**Probiotic**	LD80	Deaths	48	36	1822374
**Probiotic**	Control	Control	24	24	1487403
**Probiotic-antibiotic**	LD80	Survivors	12	4	298935
**Probiotic-antibiotic**	LD80	Deaths	36	27	1554425
**Lab line**	LD80	Survivors	24	21	1118896
**Lab line**	LD80	Deaths	12	6	311913
**Lab line–antibiotic**	LD80	Deaths	12	6	290108
**Lab line–antibiotic**	LD80	Survivors	12	6	318903

Bacterial 16S rRNA genes were partially amplified by PCR using primer pair 27F (5′-AGAGTTTGATCCTGGCTCAG-3′) and 1391R (5′-GACGGGCGGTGWGTRCA-3′) (Weisburg *et al*. 1991) to enrich microbiome DNA quantities allowing the study of individual insect gut microbiome. The products of this enrichment PCR were checked using agarose gels and deemed succesful by having sufficiently concentrated DNA of the correct amplicon size for visualisation. Critically, we must acknowledge that although necessary for downstream amplicon sequencing, enrichment PCR tends to amplify the most common fragments in an extraction. Therefore, our statistical analysis is limited to only the most abundant OTUs identified from the community. To analyse the microbial community composition, successful amplicons were exported for amplicon sequencing data (Table 1).

The targeted amplicons based on primer pair 27F-1391R were quantified *in-house* using Nanodrop (Sigma Aldrich), then frozen and shipped to the Earlham Institute (Norwich, UK) for downstream processing on Illumina MiSeq. From here, the amplicons from the first PCR were quantified using a Quant-iT™ dsDNA Assay Kit (Thermo Fisher Scientific Q33120). A second PCR was performed with the Kapa HiFi HotStart PCR kit (Roche Diagnostics 7958897001) in 50ul reactions with 20 ng of the amplicon from the first PCR, and 5ul each of an i5 and i7 Nextera XT Index kit v2 (Illumina FC-131–2001) indexed primer. After 7 cycles of PCR, the PCR products were purified with a 1x Agencourt AMPure XP bead clean up (Beckman Coulter A63882) with two 80% EtOH washes and resuspended in 25 µl of elution buffer (10 mM Tris).

The libraries were quantified using the Qubit dsDNA HS Assay Kit and sized on a PerkinElmer GX using the High Sensitivity DNA chip (PerkinElmer CLS760672). Libraries were equimolar pooled and the resulting pool was quantified by qPCR using a Kapa Library Quantification Kit (Roche Diagnostics 7960204001). The pool was diluted to 2 nM and denatured using 2 N NaOH before diluting to 20pM with Illumina HT1 buffer. The denatured pool was loaded on an Illumina MiSeq Sequencer with a 600 cycle MiSeq reagent kit v3 (Illumina MS-102–3003) at 70% loading concentration with a 20% phiX control v3 spike (Illumina FC-110-3001) as per Illumina's recommendations for low diversity amplicon sequencing.

Data were analysed in in accordance with Qiime2 (Guerrini *et al*. 2019) guidance. The Sequencing Phred scores were checked for correct encoding and the demultiplexed reads were imported. The demultiplexed reads were then summarized to allow for visualisation with Qiime2. The reads were visualized and the Illumina Amplicon sequence data was corrected and denoized, determining the values for trimming and truncation using DADA2 (Callahan *et al*. 2016). BIOM files generated were converted to human readable format (McDonald *et al*. 2012). Qiime2 was used with a pre-trained Naive Bayes classifier for classifying OTUs (Bokulich et al. 2018).

### Sequence deposition

Sequences derived from Illumina MiSeq amplicon sequencing were deposited on the NCBI Sequence Read Archive (http://trace.ncbi.nlm.nih.gov/Traces/sra) under submission **SUB9585236**.

### Statistical analysis

Analyses were performed using the *R* statistical software v3.4.2 (2018). Variation in host response to viral infection and potential effects of gut microbial supplementation on host susceptibility to viral challenge were analysed using survival analysis (Cox proportional hazards regression) in the *survival* package (Therneau and Lumley 2015).

Gut microbial community composition was analysed in *R*. Rarefaction of amplicon sequencing data increases the probability of type-II errors (McMurdie and Holmes 2014), so the data were instead normalised using the *normFactor* function in the *metagenomeSeq* package in R (Paulson *et al*. 2013).

Non-metric multidimensional scaling (NMDS) was used to analyse correlations between host microbial communities and host responses to viral infection and based on variation in the abundances of all members of the community (Wang *et al*. 2012). Here, we analysed community count data by NMDS using the *metaMDS* function. NMDS was performed using the Bray-Curtis dissimilarity index on three ordinal scales for optimal NMDS stress values in the *VEGAN* package for R (Dixon 2003). Effects of treatment group on the NMDS community clustering were tested using the *envfit* function. Community diversity indices (species number, Shannon, and Simpson diversity) were also analysed with treatment group using generalized linear models (glm).

Normalised read counts (using rarefaction) of individual members of the host gut microbiome were then analysed for potential direct roles in the host viral response using glm with (quasi-) Poisson error structure. Response variables were the read counts of microbial taxa determined by sequencing. Explanatory variables included in maximal models were: culture (lab/probiotic), viral dosage (LD80/control), antibiotic (Yes/No) and viral death (dead/survived). Extended results of all glms are presented in Supplementary materials B.

## Results

### Gut microbiome composition

#### Sequencing quality control

Sequencing of amplified DNA from caterpillar gut contents generated 9332574 raw reads with an average read length of 299 bp (274–300 bp; CV = 0.05). Post filtering, 9109934 reads were clustered to 348 distinct OTUs. For taxonomic classification and comparison, these reads were binned into their respective treatment groups (Table 1).

#### Bacterial classes

**Figure 1.**
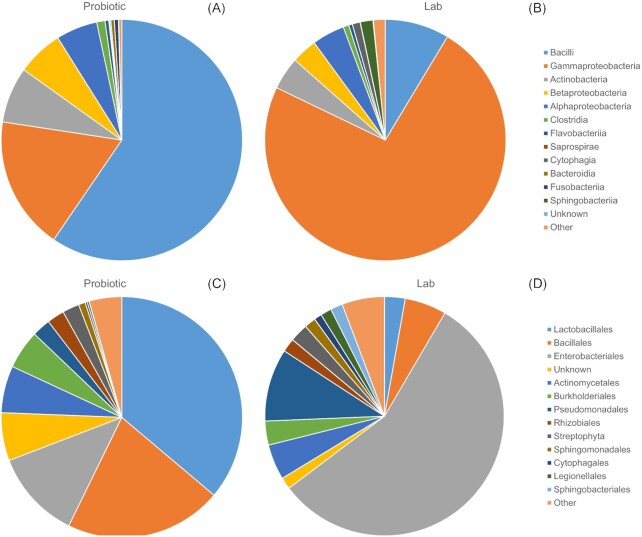
Microbial community diversity between the probiotic line and laboratory line of *S. exempta* larvae; identified to class level for (**A**) the probiotic line and (**B**) the lab line. And identified to order level for (**C**) the probiotic line and (**D**) the lab line by Illumina MiSeq.

OTUs were clustered into 28 bacterial classes, with five of these representing more than 94.7% of all the classes. The *probiotic* line harboured diverse lineages of bacterial classes (n = 23), comprising on average five classes (mean ± SD: 4.89 ± 2.16, range: 2–13) with the top five most abundant being *Bacilli, Gammaproteobacteria, Actinobacteria, Betaproteobacteria, Alphaproteobacteria* (Fig. 1A). The *lab* line harboured fewer bacterial classes (n = 15), comprising on average five classes per individual (mean ± SD: 4.51 ± 1.77, range: 2–9, Fig. 1B), dominated by *Gammaproteobacteria* (73.52%) and lacking eight classes present in the *probiotic* line (Supplementary Materials B).

#### Bacterial orders

OTUs from all treatment groups were clustered into 52 bacterial orders, with six representing more than 92.6% of all the orders. The *probiotic* line harboured bacteria belonging to 41 orders, comprising on average seven orders per individual (6.88 ± 2.98, range: 2–17) with the top 5 most abundant orders being *Lactobacillales, Bacillales, Enterobacteriales, Actinomycetales, Pseudomonadales*, and *Burkholderiales* (Fig. 1C). The *lab* line harboured 28 bacterial orders, averaging seven orders per individual larva (6.85 ± 1.78, range: 3–11; Fig. 1D) and being primarily dominated by *Enterobacteriales* (Family: *Enterobacteriaceae*, 56.28%).

#### Wild-type microbiome

A subsample of ‘wild’-type faeces accounted for 49651 of the post filtering reads. These reads were clustered into 8 bacterial classes, predominately the Bacilli (98.68%), *Actinobacteria* (0.27%) and *Gammaproteobacteria* (0.70%). Within the dominant class Bacilli, these comprised 18 orders, primarily the *Lactobacillales* (98.58%). Data on bacterial family and genera distributions are available in Supplementary Materials D, though notably during read-filtering, confident classification to genus level for many OTUs was not possible, hence are omitted here.

### Bacterial community composition interactions with treatment group

Comparing the *lab* line with the *probiotic* line allows us first to determine the efficacy of our attempted microbial manipulations. NMDS showed that a three-dimensional solution was sufficient to achieve low stress values to enable us to interpret gut community composition (stress = 0.204, Table S2).

**Figure 2.**
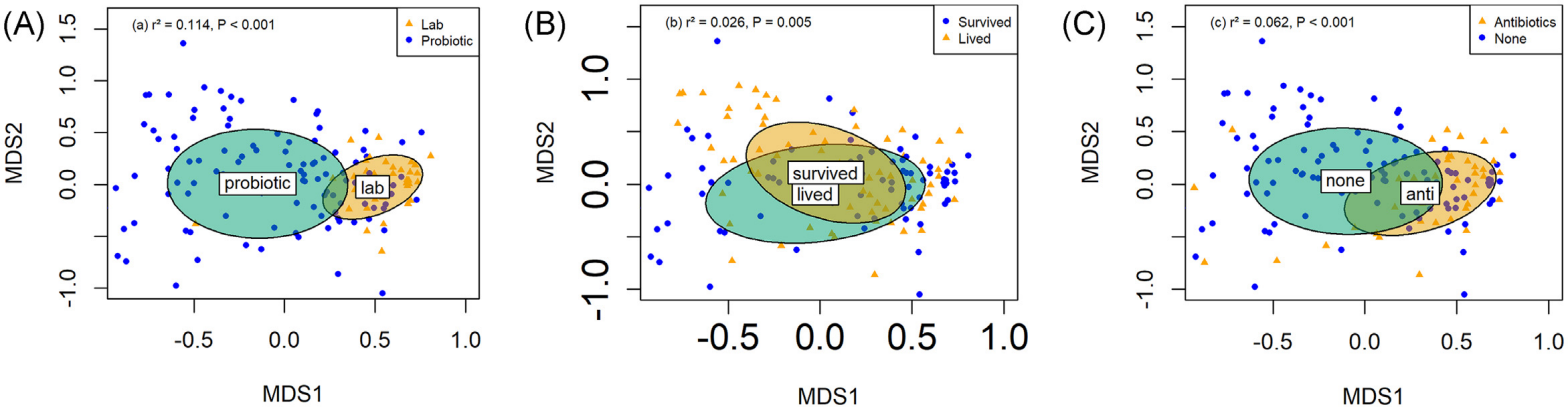
Non-metric multidimensional scaling (NMDS) surface ordination based. Clustering ellipses plotted according to *envfit* function derived centroids. (**A**) Probiotic and lab line (excluding treatment groups receiving antibiotics) gut microbiomes contain significantly different gut microbiome community structures (r² = 0.137, *P <* 0.001). (**B**) Survivors vs dead larvae following exposure to a baculovirus. (**C**) The probiotic line compared with a control probiotic sub-line that was re-exposed to dietary antibiotics.

NMDS community composition clusters were significantly correlated with treatment group, with the *probiotic* line and *lab* line forming highly distinct microbial communities, clustering in significantly different groups in the NMDS plot (r² = 0.114, *P <* 0.001; Fig. 2A). The addition of a faecal suspension to the diet for two generations of the *probiotic* line was sufficient to significantly alter the gut composition of larvae within this treatment group. Analyses of Shannon (F_1,165_ = 7.722, *P =* 0.006), and Simpson indices (F_1,165_ = 13.403, *P <* 0.001), were significantly different between the *probiotic* and *lab* treatment groups, but species number (or richness) was not (F_1,165_ = 0.131, *P =* 0.718).

Treatment groups receiving virus that survived the bioassays were significantly different from those that did not (r² = 0.026, *P =* 0.005, Fig. 2B). Species number was marginally non-significantly different between survivors and those that did not (F_1,164_ = 3.648, *P =* 0.057), but not for Shannon (F_1,164_ = 0.706, *P =* 0.401) or Simpson indices (F_1,164_ = 1.429, *P =* 0.234).

The *probiotic* line later treated with antibiotics shifted the community composition clustering significantly (r² = 0.062, *P <* 0.001, Fig. 2C). Shannon (F_1,164_ = 10.587, *P =* 0.001), and Simpson indices (F_1,163_ = 12.730, *P <* 0.001), were significantly different between the *antibiotic* treatment groups, but species number was not (F_1,165_ = 0.639, *P =* 0.428).

### Bacterial order interactions with treatment group

Four bacterial orders were the focus of statistical analysis by generalized linear models: *Bacillales, Lactobacillales, Enterobacteriales*, and *Pseudomonadales*. Combined, these orders accounted for 91% of sequence reads across the data set.

### Bacillales

**Figure 3.**
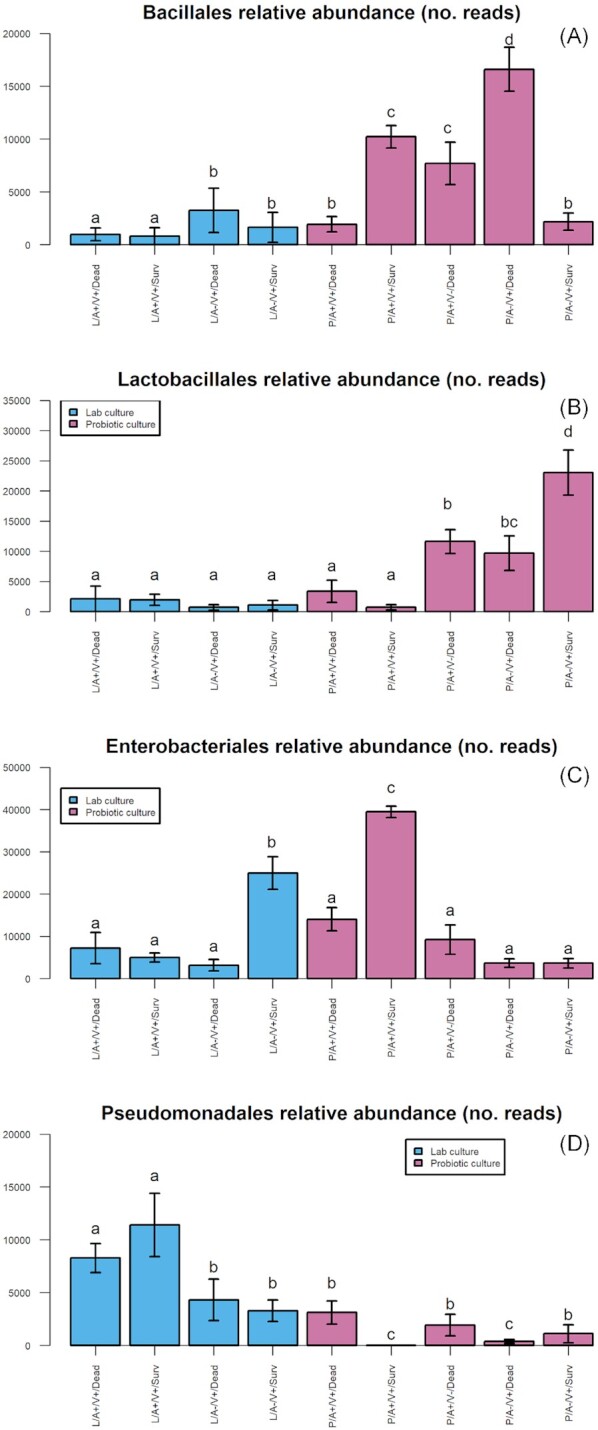
Order-level analyses of bacterial abundances from Illumina MiSeq metabarcoding data. Comprising (**A**) *Bacillales*, (**B**) *Lactobacilliales*, (**C**) *Enterobacteriales* and (**D**) *Pseudomonadales* abundance analysed between treatment groups: of **(L/P)** lab/probiotic culture, **(A+/A-)** antibiotic dosing, **(V+/V-)** viral dose and non-viral controls, and **(Dead/Surv)** LD80 survivors/dead.

The *probiotic* line was significantly enriched with *Bacillales* in comparison to the lab line (GLM: b ± SE = 1.074 ± 0.495, F_1,164_ = 14.930, *P <* 0.001; Fig. 3a). *Bacillales* were significantly reduced in abundance in individuals given antibiotic treatment (GLM: b ± SE = -1.489 ± 0.400, F_1,162_ = 19.068, *P <* 0.001; Fig. 3a). Individuals that died from viral infection had significantly higher abundance of *Bacillales* (GLM: b ± SE = 1.452 ± 0.339, F_1,164_ = 9.548, *P =* 0.024; Fig. 3a).

### Lactobacillales

The *probiotic* line was significantly enriched with *Lactobacillales* in comparison to the lab line (GLM: b ± SE = 2.262 ± 0.673, F_1,164_ = 21.726, *P <* 0.001; Fig. 3B). *Lactobacillales* were significantly reduced in abundance in individuals given antibiotic treatment (GLM: b ± SE = -1.112 ± 0.476, F_1,165_ = 16.456, *P <* 0.001; Fig. 3B). Individuals that died from viral infection had significantly lower abundance of *Lactobacillales* (GLM: b ± SE = -0.591 ± 0.275, F_1,163_ = 4.985, *P =* 0.027; Fig. 3B).

### Enterobacteriales

The *probiotic* line was significantly enriched with *Enterobacteriales* in comparison to the lab line (GLM: b ± SE = 0.498 ± 0.553, F_1,164_ = 8.755, *P =* 0.004; Fig. 3C). *Enterobacteriales* were significantly more abundant in individuals given antibiotic treatment (GLM: b ± SE = 0.694 ± 0.231, F_1,165_ = 6.083, *P =* 0.015; Fig. 3C). Individuals that died from viral infection had significantly higher abundance of *Enterobacteriales* (GLM: b ± SE = 1.581 ± 0.555, F_1,163_ = 6.755, *P =* 0.010; Fig. 3C). Finally, there was a significant interaction between culture group and the abundance of *Enterobacteriales* in the survivors of viral bioassays, with fewer *Enterobacteriales* in those that survived (GLM: b ± SE = -1.311 ± 0.609, F_1,162_ = 5.702, *P =* 0.018; Fig. 3C). The probiotic line consistently displayed increased *Enterobacteriales* read counts, whilst also having increased abundances of *Lactobacillales*, a trend which was in turn reversed by antibiotics, hence the swap over to *Enterobacteriales* here (Fig. 3C).

### Pseudomonadales

The *probiotic* line had significantly reduced *Pseudomonadales* abundance in comparison to the lab line (GLM: b ± SE = -1.250 ± 0.360, F_1,164_ = 11.646, *P <* 0.001; Fig. 3D). *Pseudomonadales* were significantly increased in abundance in individuals given antibiotic treatment (GLM: b ± SE = 0.998 ± 0.359, F_1,165_ = 8.933, *P <* 0.001; Fig. 3D). *Pseudomonadales* abundance was not significantly different between individuals that survived or died from the viral bioassay (F_1,163_ = 0.232, *P =* 0.631).

### Other

Limited significant effects were found with other orders of the gut microbiome within this experiment; full statistical results can be found in Supplementary Materials C.

### Virus bioassay

**Figure 4.**
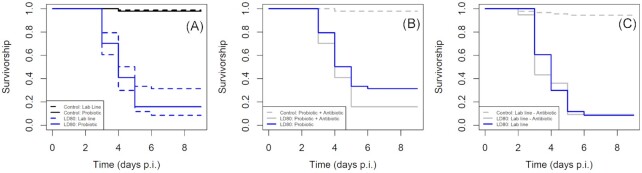
Response of *Spodoptera exempta* to Spodoptera exempta Nucleopolyhedrovirus (SpexNPV) dose varies with microbial gut supplementation. (**A**) Survival curve comparing response to LD80 dose between probiotic supplementation and lab line cultures of *S. exempta*; probiotic supplementation significantly decreases the lethality of SpexNPV. (**B**) Survival curve exploring the potentially confounding effects of genetic selection of the probiotic line. (**C**) Survival curve exploring the effects of antibiotic toxicity on susceptibility to viral infection within the lab line.

Host responses to viral infection were significantly different between gut microbiome treatments. Survival analysis showed that probiotic supplementation lowers both the overall mortality caused by SpexNPV infection, and slows down the speed of kill. An LD80 dosage was significantly more virulent to individuals in the *lab* line (*Lab*: *b* ± S.E. = 1.572 ± 0.346, z = -8.147, *P* > 0.001), than in the *probiotic* line (*Probiotic*: *b* ± S.E. = 1.992 ± 0.346, z = -6.939, *P* > 0.001), suggesting a potential protective effect of the probiotic supplementation in this infection system (Fig. 4A).

Exploring the potentially confounding effects of the experimental design, in the LD80-challenged individuals, we found no significant difference between those from the standard *lab* line and those from the *probiotic* line that had been given antibiotics (Probiotic-antibiotic: *b* ± S.E. = 4.066 ± 0.486, z = -0.675, *P =* 0.500, Fig. 4B). No difference was found between standard *lab* line individuals and *lab* line individuals that were not fed any antibiotics (Lab-non-antibiotic: *b* ± S.E. = 1.572 ± 0.026, z = -0.560, *P =* 0.570, Fig. 4C). Thus, eliminating the possibility of genetic selection and antibiotic toxicity, respectively.

The negative controls (those not challenged by the virus) were not significantly different from each other, and showed zero non-viral deaths (*Lab* line: *b* ± S.E. = 5.129 ± 0.332, z = -0.920, *P =* 0.360; Probiotic: *b* ± S.E. = 4.109 ± 0.407, z = -0.700, *P =* 0.484; Probiotic-antibiotic: *b* ± S.E. = 4.066 ± 0.486, z = -0.675, *P =* 0.500).

## Discussion

We designed this study to examine the interactions between host gut microbial symbionts and the nucleopolyhedrovirus of *Spodoptera exempta* (SpexNPV) as a model system for the interaction between gut microbiome and viral infections. Through manipulating a standardized artificial diet with the addition of field-collected frass to supplement and a broad-spectrum antibiotic (streptomycin) to reduce diversity, we successfully demonstrated the ability to manipulate and study the interaction effects of gut microbiome on a commonly occurring baculovirus of a key crop pest. Specifically, we identified that the ‘wild’-type frass was primarily dominated by members of the *Lactobacillales* (for those that could be identified to genus level, these were *Lactobacillus* spp.), whereas the lab-type larvae were dominated by *Pseudomonadales* (most commonly *Pseudomonas* sp.). Upon receiving treatment in the probiotic line, the gut community of *S. exempta* shifted away from *Enterobactereales* dominance to *Lactobacillales*. Using standardized viral bioassay techniques, we further demonstrate that this shift results in an increased resistance to SpexNPV.

We showed that an increased diversity of the gut microbiome is linked with lower viral susceptibility. This effect was consistently detected across treatment groups, and notably was lost when the gut microbiome was re-treated with a broad-spectrum antibiotic. Increased abundances of certain members of the gut microbiome (*Pseudomonadales* and *Enterobactereales*) were associated with higher viral susceptibility, suggesting possible interactions between bacteria, virus and host either increasing virulence, reducing host fitness, or co-infecting the host.

Through the use of amplicon sequencing techniques, we were able to study the abundances of bacterial classes, orders and genera present within the gut within each treatment group. Though overall results indicate that treatment groups with increased diversity have lower viral susceptibility, a more complex story emerges when looking at the orders present in bioassay survivors and those that died. When looking at the interactions with certain bacterial classes, our results suggest that the *Lactobacillales* increase survival of their host to a viral challenge.

### Gut microbiome affects host resistance to parasites

Through our experimental manipulation of an artificial diet, we have successfully demonstrated a link between gut bacterial diversity and susceptibility to virus. We have not directly demonstrated a mode-of-action for this interaction, but the importance of the microbial community may result from the complementary and synergistic antiparasitic effects of different microbes (Prigot-Maurice *et al*. 2022). The data we collected on the gut microbiome of field-located *S. exempta* larvae, and data from previous studies (Graham *et al*. 2012) clearly demonstrates a decline in gut microbial diversity in lab stocks kept for over 70-generations. This decline in gut-microbial diversity is linked with an increase in suseceptibility to the nucleopolyhedrovirus pathogen. The benefits of a diverse microbial community are widely accepted in mammalian and human biology especially in regards to resilience to pathogens (Zheng et al. 2020), the mechanisms of protection are poorly understood in animal models (Kešnerová *et al*. 2017). Potential mechanisms include high functional diversity (Carrara et al. 2015), increased functional redundancies (Moya and Ferrer 2016), and metabolic cross-feeding (Hoek and Merks 2017).

Both abiotic and biotic factors can affect host resistance to parasites. Host diet and host gut microbiomes are two increasingly recognized factors influencing disease resistance (Vogel *et al*. 2018, Hammer *et al*. 2019). We are only just beginning to understand the role of gut microbiome as a superorganism; the role of the ‘holobiont’ organism (Douglas and Werren 2016) in resistance to infection has had limited empirical or manipulative study (Harris *et al*. 2019, Desselberger 2020, Almire *et al*. 2021). A diverse bee gut community is protective against the bacterial pathogen *Paenibacillius larvae*, the causative agent of American foulbrood (Alippi and Reynaldi 2006, Forsgren *et al*. 2010). Desert locusts also have decreased pathogen colonization with increased numbers of gut bacterial species (Dillon *et al*. 2005). And a diverse gut microbiome theoretically stimulates antimicrobial peptide production (a key aspect of insect immune systems) in the black soldier fly (Vogel *et al*. 2018).

Studies have shown separately that diet affects the gut microbiome and that the gut microbiome affects parasitic resistance in both mice and mosquitoes infected with *Plasmodium* spp. (Linenberg *et al*. 2016, Villarino *et al*. 2016). The ‘core’ microbiome present in social Hymenoptera, such as bumblebees, have also been a focus for pathological resistance provided to hosts (Praet *et al*. 2018). Host immunity plays a key role in both directly and indirectly modulating diet–microbiome–disease interactions, particularly given the emerging evidence for ‘immune priming’ by microbial symbionts in arthropods (Sansone *et al*. 2015, Emery *et al*.^2017^). Similarly, manipulation of honeybees’ diets decreased relative abundance of *Frischella perrara*, and other microsporidian parasites; whether this increased resistance is the result of a diet-altered microbiome is unknown (Maes *et al*. 2016).

### A potential role for the lactobacillales in antiviral symbiosis

The results from our present study suggest that individual *S. exempta* larvae with a greater abundance of *Lactobacilli* in their gut are more resistant to *SpexNPV*. As well as simply reducing viral susceptibility in the *probiotic* culture line, we found that this effect was reversed with an additional antibiotic treatment (which reduced the abundance *Lactobacilli*).

Though our results provide some limited evidence for the role of the Lactobacilli in decreasing viral susceptibility in *S. exempta*, evidence from other invertebrate studies suggest that it is far more likely that the combined community present in the gut may have a more important role. For example, in honey bees, eleven cultured bacterial phylotypes differentially inhibit the growth of the bacterial pathogen *Paenibacillius larvae* in vitro, but only the microbial cocktail of all 11 bacterial phylotypes completely inhibits the growth of *P. larvae* in vitro and in vivo (Yoshiyama and Kimura 2009).

There is some limited evidence in other systems, for example the *Aedes aegypti*-Zika virus system, of viruses impacting the gut microbiome (Villegas et al. 2018). Some pathogens may retroactively impact the gut microbiome of their host, for example the gut microbial community of the grain beetle (*Tenebrio molitor*) is altered following parasitism by the tapeworm *Hymenolepis diminuta* (Fredensborg *et al*. 2020). Critically, within our study we did not observe any significant interactions between gut bacterial composition and exposure to SpexNPV in the bioassay, when controlling for the outcome of these bioassays. This means that we did not observe any impact of the virus on the gut microbiome of *S. exempta* within this study.

Specific microbial symbionts can play important roles in animal health, particularly in mitigating infectious diseases. For example, aphids harbour non-gut-associated bacterial symbionts (*Buchnera*) that protect them against fungal pathogens and parasitoid wasps (Scarborough *et al*. 2005, Vorburger *et al*. 2010). Similarly, beewolf wasps incorporate symbiotic bacteria into their larval cocoons for protection against pathogenic fungi (Kaltenpoth *et al*.^2005^, Koehler*et al*. 2013). Though other studies have used similar methodologies to identify bacterial taxa to genus or species level and associate more specific interactions between microbial actors (Fitzpatrick *et al*. 2018), taxonomic assignment from read lengths of 299 bp is disingenuous, therefore limiting the ability of this study to draw further conclusions. It is clear that gut-associated microbial symbionts play major roles in infectious disease dynamics, with changes in microbial community structure and function being correlated with parasite infection in several systems. Further study could usefully interrogate the roles *in-silico* or interactions *in-vitro* of sufficiently identified species in the gut of *S. exempta*.

### The presence of *pseudomonadales* and *enterobactereales* in more susceptible individuals

We found evidence of a small amount of *Enterobactereales* enrichment in the probiotic group and higher abundances of *Pseudomonadales* in the lab group. These bacterial orders were also increased in abundance in the antibiotic-control group. Through the combination of bacterial supplementation and viral bioassays, we have demonstrated consequently that both the *Pseudomonadales* and the *Enterobactereales* were significantly more abundant in groups that had significantly increased susceptibility to SpexNPV. Immunological research is beginning to understand the dynamics of co-infection between bacteria and viruses (Smith *et al*. 2013).

Studies of the interaction between bacteria and H1N1 Influenza virus critically focus on *Strepococcus*, a member of the *Enterobactereales* (Palacios *et al*. 2009). The synergistic infection between *Enterobactereales* and SpexNPV we have demonstrated here highlights the need for further study into the interaction between these bacteria and viruses on a broader community microbial community level. Furthermore, the replicability and robustness of the *S. exempta*—SpexNPV—gut microbiome system may serve as an important model system for the study of both symbiosis and coinfection dynamics with host viral infections.

### Final remarks

Gut bacterial diversity, leading to high functional diversity (Carrara *et al*. 2015), increased functional redundancies (Moya and Ferrer 2016), or metabolic cross-feeding (Hoek and Merks 2017) results in an observable resistance to viral infection. Our study has demonstrated an observable interaction between increasing gut bacterial diversity and reduced susceptibility to viral infection.

Some studies have suggested there is no resident gut microbiome for caterpillars due to the physical structure of their digestive systems (Hammer *et al*. 2017). Though notably, this claim remains controversial within the field (Voirol *et al*. 2018, Hammer *et al*. 2019). The results of our study clearly demonstrate an interaction between microbial symbionts sourced from caterpillar faeces and a viral pathogen that infects its host through the gut lining.

The results we present have significant implications for running long-term experiments on insect cultures with a long captivity time. Though the appreciation of the difference between a ‘wild type’ and a ‘lab type’ is well known, and the need to acknowledge this effect when performing bioassays, the causative relationship due to suppression of a naturally occurring gut microbiome through standard laboratory protocols (2021) is an important and novel result that will have widespread impacts on viral pathogen studies. These findings may go some way towards explaining widespread result differentials between lab experiments and field trials of biopesticides (Darriet *et al*. 2010, Behle and Popham 2012, Amoabeng *et al*. 2014).

Our results demonstrate the ability to alter the gut microbiome of an insect crop pest, and the significant impacts of this on the outcome of a viral bioassay. Widespread application of SpexNPV as a biopesticide could provide a viable alternative to chemical control for armyworm control in Africa (Grzywacz *et al*. 2008). The synergistic effects of *Pseudomonadales* and the *Enterobactereales* on the viral bioassays may have substantial potential as a ‘cocktail’ biopesticide.

## Supplementary Material

fiac147_Supplemental_Files
