# Supplementary material for: Gut microbial community supplementation and reduction modulates African armyworm susceptibility to a baculovirus
Source: FEMS Microbiol Ecol. 2022 Dec 6;99(1):fiac147. doi: 10.1093/femsec/fiac147 (PMC9764207; doi:10.1093/femsec/fiac147)
Supplement: fiac147_Supplemental_Files [file fiac147_supplemental_files.zip › Supp_data_A.docx]

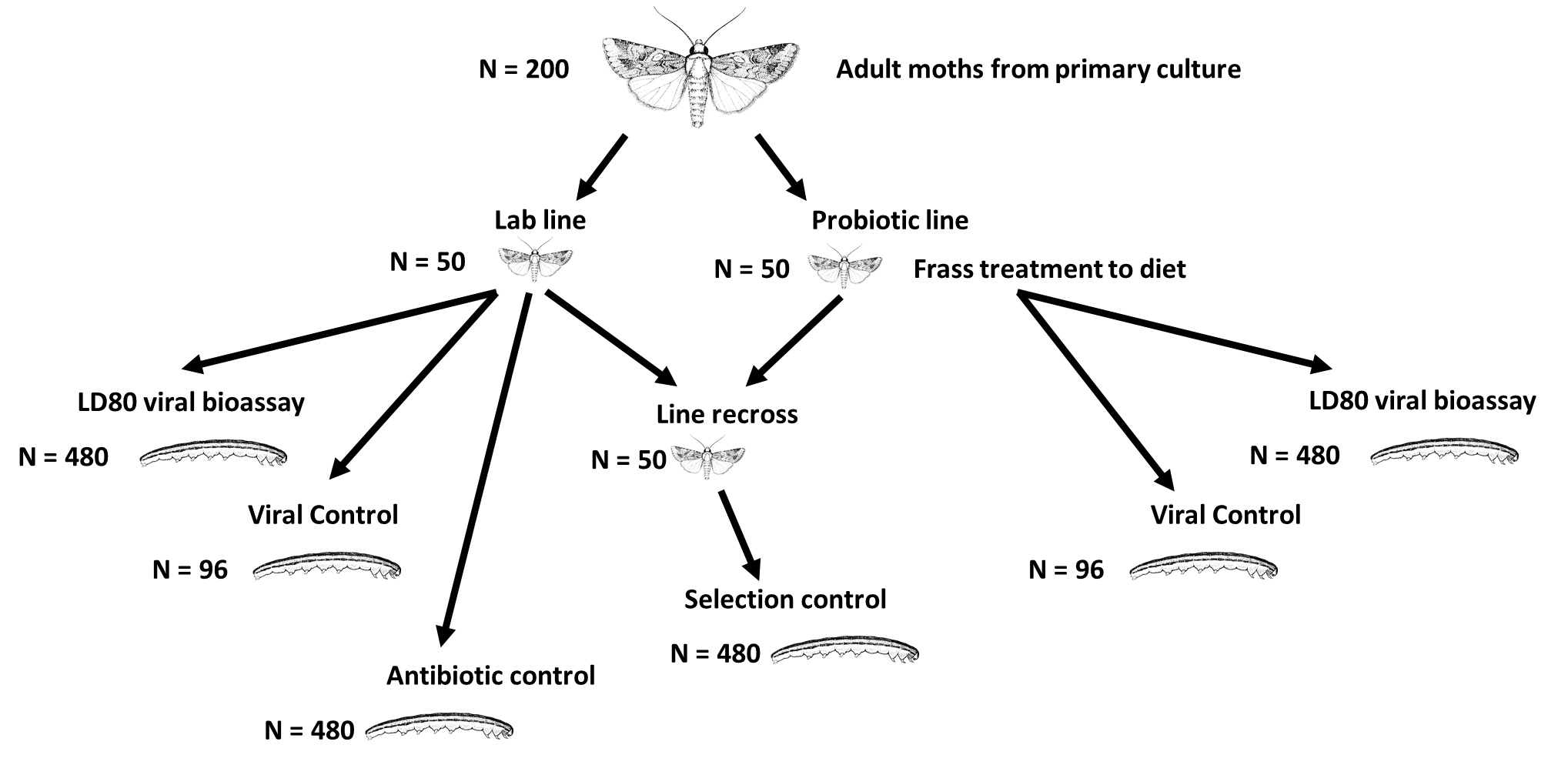


**Supplementary materials A.** Schematic diagram of the breeding program for each treatment group within the experiment. All larvae are derived from the original genetically diverse primary culture maintained by the laboratory group. Each line was maintained by crossing moths with known genetically diverse heritage in order to maintain overall culture diversity. Individuals for each treatment group were selected from the Lab line and Probiotic line respectively. Individual larvae were randomly selected from offspring of each crossbred family line throughout the duration of the experiment (i.e. Family A & Family B = Offspring AB). A studbook of these crosses was maintained to prevent back-crossing within generations; an equal number of larvae were selected for each experiment from across all families within the lab and probiotic lines respectively.
