# Supplementary material for: Gut microbial community supplementation and reduction modulates African armyworm susceptibility to a baculovirus
Source: FEMS Microbiol Ecol. 2022 Dec 6;99(1):fiac147. doi: 10.1093/femsec/fiac147 (PMC9764207; doi:10.1093/femsec/fiac147)
Supplement: fiac147_Supplemental_Files [file fiac147_supplemental_files.zip › Supp_data_B.docx]

**Table 1**. Data analyses of order-level classification gut microbiome.

**Species number**

**Maximal model:**

glm(formula = A ~ Culture * Dead * Viral.Dose + Antibiotic, family = "quasipoisson",

data = p6, na.action = "na.fail")

Deviance Residuals:

Min 1Q Median 3Q Max

-3.6605 -1.5287 -0.5618 0.6617 16.1143

|  | **b** | **± S.E.** | **F** | **P** | **Significance** |
| --- | --- | --- | --- | --- | --- |
| (Intercept) | 2.69466 | 0.311366 |  |  |  |
| **Culture** | -0.01254 | 0.230037 | **0.1298** | **0.71913** |  |
| Dead | -0.25926 | 0.267282 | 3.6237 | 0.05875 | . |
| Viral.Dose | 0.041839 | 0.212665 | 0.0266 | 0.87075 |  |
| Antibiotic | -0.0021 | 0.146544 | 0.0001 | 0.99187 |  |
| Culture:Dead | 0.050165 | 0.307516 | 0.0265 | 0.8708 |  |

Null deviance: 874.12 on 166 degrees of freedom

Residual deviance: 842.95 on 161 degrees of freedom

**Most parsimonious model:**

glm(formula = A ~ Culture + Dead, family = "quasipoisson",

data = p6)

Deviance Residuals:

Min 1Q Median 3Q Max

-3.8781 -1.6653 -0.6531 0.7142 15.0835

|  | **b** | **± S.E.** | **F** | **P** | **Significance** |
| --- | --- | --- | --- | --- | --- |
| (Intercept) | 2.63384 | 0.14934 |  |  |  |
| Culture | 0.05877 | 0.13854 | 0.1307 | 0.71822 |  |
| Dead | -0.2325 | 0.121689 | 3.6482 | 0.05788 | . |

Null deviance: 874.12 on 166 degrees of freedom

Residual deviance: 868.27 on 164 degrees of freedom

**Shannon Index**

**Maximal model:**

glm(formula = H ~ Culture * Dead * Viral.Dose + Antibiotic, family = "quasipoisson",

data = p6, na.action = "na.fail")

Deviance Residuals:

Min 1Q Median 3Q Max

-1.44479 -0.53118 -0.00199 0.48311 1.79674

|  | **b** | **± S.E.** | **F** | **P** | **Significance** |
| --- | --- | --- | --- | --- | --- |
| (Intercept) | 2.6259 | 0.2771 |  |  |  |
| Culture | **-0.5991** | **0.2166** | 7.8077 | 0.005834 | ** |
| Dead | -0.3996 | 0.2412 | 4.1958 | 0.042148 | * |
| Viral.Dose | -0.4154 | 0.177 | 5.7472 | 0.01766 | * |
| Antibiotic | -0.3838 | 0.1329 | 8.214 | 0.004713 | ** |
| Culture:Dead | 0.2394 | 0.2754 | 0.7555 | 0.386048 |  |

Null deviance: 88.576 on 166 degrees of freedom

Residual deviance: 75.968 on 161 degrees of freedom

AIC: 356.38

**Most parsimonious model:**

glm(formula = H ~ Culture + Antibiotic + Dead + Viral.Dose +

Culture:Viral.Dose, data = p6)

Deviance Residuals:

Min 1Q Median 3Q Max

-1.44479 -0.55312 0.04842 0.42878 1.83277

|  | **b** | **± S.E.** | **F** | **P** | **Significance** |
| --- | --- | --- | --- | --- | --- |
| (Intercept) | 2.5363 | 0.2571 |  |  |  |
| Culture | -0.4516 | 0.1344 | 7.8195 | 0.005794 | ** |
| Antibiotic | -0.3808 | 0.1328 | 10.5871 | 0.001386 | ** |
| Dead | -0.2212 | 0.1265 | 0.7061 | 0.40197 |  |
| Viral.Dose | -0.4514 | 0.172 | 6.8912 | 0.009491 | ** |

Null deviance: 88.576 on 166 degrees of freedom

Residual deviance: 76.324 on 162 degrees of freedom

AIC: 355.16

**Simpson Index**

**Maximal model:**

glm(formula = S ~ Culture * Dead * Viral.Dose + Antibiotic, data = p6,

na.action = "na.fail")

Deviance Residuals:

Min 1Q Median 3Q Max

-0.67174 -0.16058 0.03744 0.17052 0.42903

|  | **b** | **± S.E.** | **F** | **P** | **Significance** |
| --- | --- | --- | --- | --- | --- |
| (Intercept) | 1.11952 | 0.09912 |  |  |  |
| Culture | -0.22504 | 0.07746 | 1.33E+01 | 0.000353 | *** |
| Dead | -0.07503 | 0.08626 | 1.4223 | 0.234785 |  |
| Viral.Dose | -0.19767 | 0.06332 | 9.09E+00 | 0.002981 | ** |
| Antibiotic | -0.18199 | 0.04755 | 14.5753 | 0.000192 | *** |
| Culture:Dead | 0.04331 | 0.09851 | 0.1933 | 0.660766 |  |

Null deviance: 12.0501 on 166 degrees of freedom

Residual deviance: 9.7192 on 161 degrees of freedom

AIC: 12.996

**Most parsimonious model:**

glm(formula = S ~ Culture + Dead + Antibiotic + Viral.Dose, data = p6)

Deviance Residuals:

Min 1Q Median 3Q Max

-0.6717 -0.1661 0.0354 0.1652 0.4355

|  | **b** | **± S.E.** | **F** | **P** | **Significance** |
| --- | --- | --- | --- | --- | --- |
| (Intercept) | 1.10332 | 0.09179 |  |  |  |
| Culture | -0.19834 | 0.04799 | 13.3943 | 0.000341 | *** |
| Dead | -0.04275 | 0.04516 | 1.4294 | 0.233612 |  |
| Antibiotic | -0.18146 | 0.04741 | 12.7303 | 0.000474 | *** |
| Viral.Dose | -0.20419 | 0.0614 | 11.0574 | 0.001093 | ** |

Null deviance: 12.0501 on 166 degrees of freedom

Residual deviance: 9.7309 on 162 degrees of freedom

**Table 2**. Data analyses of order-level classification gut microbiome.

| **Lactobacillales** | Group | Mean | Stdev | Min | Max | Median | b | ± SE | t | P | Significance |
| --- | --- | --- | --- | --- | --- | --- | --- | --- | --- | --- | --- |
|  | labantild80dead | 2122 | 5150 | 0 | 12635 | 16 | 1.282 | 0.215 | 5.951 | 0.000 | *** |
|  | labantild80surv | 1982 | 2259 | 7 | 4934 | 1411 | 0.418 | 0.277 | 1.507 | 0.134 |  |
|  | labld80dead | 704 | 1087 | 15 | 2515 | 37 | 0.270 | 0.286 | 0.942 | 0.348 |  |
|  | labld80surv | 1081 | 3604 | 7 | 15495 | 21 | 0.026 | 0.244 | 0.107 | 0.915 |  |
|  | probioticantibioticdead | 3362 | 9518 | 0 | 47181 | 34 | 0.282 | 0.233 | 1.209 | 0.228 |  |
|  | probioticantibioticsurv | 730 | 914 | 23 | 1957 | 470 | 0.392 | 0.306 | 1.283 | 0.201 |  |
|  | probioticcontrolsurv | 11615 | 9588 | 1020 | 31443 | 9275 | 0.910 | 0.226 | 4.027 | 0.000 | *** |
|  | probioticld80dead | 9706 | 17253 | 22 | 62460 | 1324 | 0.682 | 0.224 | 3.041 | 0.003 | ** |
|  | probioticld80surv | 23036 | 22612 | 18 | 70890 | 15542 | 0.864 | 0.223 | 3.879 | 0.000 | *** |
|  |  |  |  |  |  |  |  |  |  |  |  |
| **Enterobacteriales** | **Group** | **Mean** | **Stdev** | **Min** | **Max** | **Median** | **b** | **± SE** | **t** | **P** |  |
|  | labantild80dead | 7203 | 9044 | 12 | 24098 | 4985 | 8.882 | 0.567 | 15.674 | 0.000 | *** |
|  | labantild80surv | 4985 | 2631 | 10 | 6836 | 6157 | -0.368 | 0.886 | -0.415 | 0.678 |  |
|  | labld80dead | 3160 | 3266 | 25 | 8774 | 2089 | -0.824 | 1.026 | -0.803 | 0.423 |  |
|  | labld80surv | 24952 | 17548 | 3864 | 61256 | 22444 | 1.243 | 0.590 | 2.107 | 0.037 | * |
|  | probioticantibioticdead | 14033 | 14320 | 10 | 56408 | 8970 | 0.667 | 0.598 | 1.115 | 0.267 |  |
|  | probioticantibioticsurv | 39435 | 2671 | 36496 | 42955 | 39144 | 1.700 | 0.640 | 2.658 | 0.009 | ** |
|  | probioticcontrolsurv | 9234 | 16935 | 0 | 74340 | 960 | 0.248 | 0.620 | 0.401 | 0.689 |  |
|  | probioticld80dead | 3686 | 5936 | 0 | 22123 | 385 | -0.670 | 0.653 | -1.027 | 0.306 |  |
|  | probioticld80surv | 3634 | 6636 | 0 | 28309 | 39 | -0.684 | 0.651 | -1.050 | 0.295 |  |
|  |  |  |  |  |  |  |  |  |  |  |  |
| **Pseudomonadales** | **Group** | **Mean** | **Stdev** | **Min** | **Max** | **Median** | **b** | **± SE** | **t** | **P** |  |
|  | labantild80dead | 8284 | 3367 | 5223 | 12439 | 7563 | 2.192 | 0.297 | 7.370 | 0.000 | *** |
|  | labantild80surv | 11400 | 7359 | 4331 | 24405 | 9290 | 0.025 | 0.418 | 0.060 | 0.952 |  |
|  | labld80dead | 4320 | 4823 | 0 | 11883 | 2837 | -0.450 | 0.477 | -0.944 | 0.347 |  |
|  | labld80surv | 3282 | 4707 | 0 | 13690 | 1014 | -0.540 | 0.363 | -1.487 | 0.139 |  |
|  | probioticantibioticdead | 3128 | 5695 | 0 | 25953 | 20 | -0.735 | 0.360 | -2.043 | 0.043 | * |
|  | probioticantibioticsurv | 20 | 19 | 0 | 41 | 20 | -1.334 | 0.769 | -1.734 | 0.085 | . |
|  | probioticcontrolsurv | 1928 | 4958 | 0 | 15619 | 0 | -1.684 | 0.456 | -3.697 | 0.000 | *** |
|  | probioticld80dead | 375 | 1139 | 0 | 6064 | 0 | -1.458 | 0.390 | -3.743 | 0.000 | *** |
|  | probioticld80surv | 1117 | 5213 | 0 | 31200 | 0 | -2.224 | 0.470 | -4.731 | 0.000 | *** |
|  |  |  |  |  |  |  |  |  |  |  |  |
| **Bacillales** | **Group** | **Mean** | **Stdev** | **Min** | **Max** | **Median** | **b** | **± SE** | **t** | **P** |  |
|  | labantild80dead | 984 | 1472 | 0 | 3803 | 500 | 1.421 | 0.343 | 4.145 | 0.000 | *** |
|  | labantild80surv | 806 | 1966 | 0 | 4819 | 0 | -0.798 | 0.615 | -1.297 | 0.197 |  |
|  | labld80dead | 3252 | 5139 | 0 | 11396 | 9 | -0.157 | 0.505 | -0.312 | 0.756 |  |
|  | labld80surv | 1662 | 6495 | 0 | 29803 | 18 | -0.339 | 0.406 | -0.834 | 0.405 |  |
|  | probioticantibioticdead | 1944 | 3774 | 0 | 14433 | 17 | -0.093 | 0.382 | -0.243 | 0.809 |  |
|  | probioticantibioticsurv | 10219 | 2130 | 8052 | 12855 | 9985 | 0.800 | 0.444 | 1.803 | 0.073 | . |
|  | probioticcontrolsurv | 7698 | 9815 | 0 | 31220 | 2630 | 0.420 | 0.370 | 1.136 | 0.258 |  |
|  | probioticld80dead | 16605 | 12474 | 0 | 36432 | 13211 | 0.719 | 0.357 | 2.017 | 0.045 | * |
|  | probioticld80surv | 2187 | 4914 | 0 | 25302 | 8 | -0.151 | 0.374 | -0.405 | 0.686 |  |
|  |  |  |  |  |  |  |  |  |  |  |  |
| **Actinomycetales** | **Group** | **Mean** | **Stdev** | **Min** | **Max** | **Median** | **b** | **± SE** | **t** | **P** |  |
|  | labantild80dead | 961 | 1489 | 0 | 3487 | 85 | 1.251 | 0.339 | 3.694 | 0.000 | *** |
|  | labantild80surv | 2420 | 4879 | 0 | 12196 | 0 | -0.200 | 0.505 | -0.396 | 0.693 |  |
|  | labld80dead | 4144 | 3689 | 145 | 10920 | 3468 | 0.801 | 0.408 | 1.966 | 0.051 | . |
|  | labld80surv | 1018 | 1868 | 0 | 7834 | 115 | 0.335 | 0.372 | 0.900 | 0.369 |  |
|  | probioticantibioticdead | 2839 | 4450 | 0 | 15537 | 498 | 0.394 | 0.363 | 1.084 | 0.280 |  |
|  | probioticantibioticsurv | 4641 | 6211 | 0 | 13567 | 2499 | 0.566 | 0.461 | 1.229 | 0.221 |  |
|  | probioticcontrolsurv | 4771 | 8288 | 0 | 37265 | 1176 | 0.516 | 0.363 | 1.423 | 0.157 |  |
|  | probioticld80dead | 1562 | 3292 | 0 | 16665 | 148 | 0.336 | 0.358 | 0.939 | 0.349 |  |
|  | probioticld80surv | 2356 | 6285 | 0 | 36980 | 249 | 0.220 | 0.360 | 0.610 | 0.543 |  |
|  |  |  |  |  |  |  |  |  |  |  |  |
| **Burkholderiales** | **Group** | **Mean** | **Stdev** | **Min** | **Max** | **Median** | **b** | **± SE** | **t** | **P** |  |
|  | labantild80dead | 1046 | 1832 | 0 | 4750 | 352 | 1.657 | 0.370 | 4.477 | 0.000 | *** |
|  | labantild80surv | 736 | 1804 | 0 | 4418 | 0 | -1.322 | 0.807 | -1.638 | 0.103 |  |
|  | labld80dead | 4086 | 6330 | 0 | 12296 | 0 | -0.514 | 0.605 | -0.849 | 0.397 |  |
|  | labld80surv | 323 | 991 | 0 | 4442 | 0 | -1.022 | 0.496 | -2.060 | 0.041 | * |
|  | probioticantibioticdead | 4327 | 10707 | 0 | 50100 | 2 | -0.403 | 0.427 | -0.942 | 0.347 |  |
|  | probioticantibioticsurv | 1882 | 3739 | 0 | 7490 | 18 | -0.358 | 0.657 | -0.546 | 0.586 |  |
|  | probioticcontrolsurv | 4532 | 8977 | 0 | 31503 | 7 | -0.437 | 0.436 | -1.002 | 0.318 |  |
|  | probioticld80dead | 352 | 1226 | 0 | 6777 | 11 | -0.676 | 0.427 | -1.584 | 0.115 |  |
|  | probioticld80surv | 2482 | 8629 | 0 | 47746 | 0 | -0.754 | 0.429 | -1.757 | 0.081 | . |
|  |  |  |  |  |  |  |  |  |  |  |  |
| **Streptophyta** | **Group** | **Mean** | **Stdev** | **Min** | **Max** | **Median** | **b** | **± SE** | **t** | **P** |  |
|  | labantild80dead | 4098 | 6522 | 0 | 14704 | 10 | 1.369 | 0.517 | 2.646 | 0.009 | ** |
|  | labantild80surv | 2394 | 2786 | 0 | 6415 | 1755 | 0.072 | 0.719 | 0.100 | 0.921 |  |
|  | labld80dead | 0 | 0 | 0 | 0 | 0 | -16.671 | 1308.219 | -0.013 | 0.990 |  |
|  | labld80surv | 1140 | 4597 | 0 | 21007 | 0 | -1.526 | 0.787 | -1.940 | 0.054 | . |
|  | probioticantibioticdead | 1633 | 7159 | 0 | 36816 | 0 | -1.365 | 0.707 | -1.929 | 0.055 | . |
|  | probioticantibioticsurv | 15 | 30 | 0 | 59 | 0 | -1.345 | 1.345 | -1.000 | 0.319 |  |
|  | probioticcontrolsurv | 2114 | 4777 | 0 | 18541 | 0 | -0.647 | 0.629 | -1.029 | 0.305 |  |
|  | probioticld80dead | 785 | 2489 | 0 | 12156 | 0 | -0.831 | 0.608 | -1.367 | 0.174 |  |
|  | probioticld80surv | 369 | 1475 | 0 | 8512 | 0 | -1.376 | 0.663 | -2.075 | 0.040 | * |
|  |  |  |  |  |  |  |  |  |  |  |  |
| **Rhizobiales** | **Group** | **Mean** | **Stdev** | **Min** | **Max** | **Median** | **b** | **± SE** | **t** | **P** |  |
|  | labantild80dead | 0 | 0 | 0 | 0 | 0 | -17.300 | 2991.000 | -0.006 | 0.995 |  |
|  | labantild80surv | 102 | 250 | 0 | 612 | 0 | 17.370 | 2991.000 | 0.006 | 0.995 |  |
|  | labld80dead | 2114 | 4573 | 0 | 11422 | 190 | 18.610 | 2991.000 | 0.006 | 0.995 |  |
|  | labld80surv | 247 | 962 | 0 | 4415 | 0 | 17.440 | 2991.000 | 0.006 | 0.995 |  |
|  | probioticantibioticdead | 0 | 0 | 0 | 0 | 0 | 0.000 | 3307.000 | 0.000 | 1.000 |  |
|  | probioticantibioticsurv | 17 | 35 | 0 | 69 | 0 | 17.360 | 2991.000 | 0.006 | 0.995 |  |
|  | probioticcontrolsurv | 2574 | 4357 | 0 | 14364 | 0 | 18.530 | 2991.000 | 0.006 | 0.995 |  |
|  | probioticld80dead | 56 | 182 | 0 | 869 | 0 | 17.370 | 2991.000 | 0.006 | 0.995 |  |
|  | probioticld80surv | 842 | 3721 | 0 | 20200 | 0 | 17.000 | 2991.000 | 0.006 | 0.995 |  |
|  |  |  |  |  |  |  |  |  |  |  |  |
| **Sphingomonadales** | **Group** | **Mean** | **Stdev** | **Min** | **Max** | **Median** | **b** | **± SE** | **t** | **P** |  |
|  | labantild80dead | 496 | 892 | 0 | 2204 | 0 | 0.872 | 0.563 | 1.550 | 0.123 |  |
|  | labantild80surv | 737 | 1804 | 0 | 4419 | 0 | -0.536 | 0.926 | -0.579 | 0.563 |  |
|  | labld80dead | 430 | 1054 | 0 | 2582 | 0 | -0.603 | 0.946 | -0.637 | 0.525 |  |
|  | labld80surv | 591 | 1934 | 0 | 7857 | 0 | -0.879 | 0.731 | -1.203 | 0.231 |  |
|  | probioticantibioticdead | 0 | 0 | 0 | 0 | 0 | -18.175 | 1422.348 | -0.013 | 0.990 |  |
|  | probioticantibioticsurv | 2793 | 5548 | 0 | 11115 | 28 | 0.334 | 0.810 | 0.412 | 0.681 |  |
|  | probioticcontrolsurv | 0 | 1 | 0 | 6 | 0 | -3.384 | 1.628 | -2.079 | 0.039 | * |
|  | probioticld80dead | 52 | 224 | 0 | 1301 | 0 | -1.281 | 0.712 | -1.800 | 0.074 | . |
|  | probioticld80surv | 958 | 5781 | 0 | 35172 | 0 | -1.369 | 0.720 | -1.902 | 0.059 | . |
|  |  |  |  |  |  |  |  |  |  |  |  |
| **Clostridiales** | **Group** | **Mean** | **Stdev** | **Min** | **Max** | **Median** | **b** | **± SE** | **t** | **P** |  |
|  | labantild80dead | 210 | 514 | 0 | 1258 | 0 | 0.174 | 0.921 | 0.189 | 0.851 |  |
|  | labantild80surv | 0 | 0 | 0 | 0 | 0 | -16.476 | 2112.612 | -0.008 | 0.994 |  |
|  | labld80dead | 0 | 0 | 0 | 0 | 0 | -16.476 | 2112.612 | -0.008 | 0.994 |  |
|  | labld80surv | 342 | 1520 | 0 | 6972 | 0 | -0.339 | 1.090 | -0.311 | 0.757 |  |
|  | probioticantibioticdead | 422 | 1520 | 0 | 5950 | 0 | -0.619 | 1.095 | -0.566 | 0.572 |  |
|  | probioticantibioticsurv | 22 | 44 | 0 | 87 | 0 | -0.061 | 1.483 | -0.041 | 0.967 |  |
|  | probioticcontrolsurv | 385 | 1882 | 0 | 9218 | 0 | -0.900 | 1.170 | -0.769 | 0.443 |  |
|  | probioticld80dead | 252 | 824 | 0 | 4397 | 0 | 0.187 | 0.982 | 0.190 | 0.849 |  |
|  | probioticld80surv | 517 | 1634 | 0 | 7730 | 0 | 0.122 | 0.985 | 0.124 | 0.902 |  |
|  |  |  |  |  |  |  |  |  |  |  |  |
| **Flavobacteriales** | **Group** | **Mean** | **Stdev** | **Min** | **Max** | **Median** | **b** | **± SE** | **t** | **P** |  |
|  | labantild80dead | 0 | 0 | 0 | 0 | 0 | -16.300 | 1951.000 | -0.008 | 0.993 |  |
|  | labantild80surv | 262 | 642 | 0 | 1573 | 0 | 16.510 | 1951.000 | 0.008 | 0.993 |  |
|  | labld80dead | 0 | 0 | 0 | 0 | 0 | 0.000 | 2759.000 | 0.000 | 1.000 |  |
|  | labld80surv | 220 | 756 | 0 | 3433 | 0 | 16.470 | 1951.000 | 0.008 | 0.993 |  |
|  | probioticantibioticdead | 1377 | 3635 | 0 | 15016 | 0 | 17.280 | 1951.000 | 0.009 | 0.993 |  |
|  | probioticantibioticsurv | 78 | 155 | 0 | 310 | 0 | 16.660 | 1951.000 | 0.009 | 0.993 |  |
|  | probioticcontrolsurv | 112 | 550 | 0 | 2693 | 0 | 15.320 | 1951.000 | 0.008 | 0.994 |  |
|  | probioticld80dead | 327 | 988 | 0 | 4879 | 0 | 16.900 | 1951.000 | 0.009 | 0.993 |  |
|  | probioticld80surv | 51 | 239 | 0 | 1429 | 0 | 15.580 | 1951.000 | 0.008 | 0.994 |  |
