# Supplementary material for: Gut microbial community supplementation and reduction modulates African armyworm susceptibility to a baculovirus
Source: FEMS Microbiol Ecol. 2022 Dec 6;99(1):fiac147. doi: 10.1093/femsec/fiac147 (PMC9764207; doi:10.1093/femsec/fiac147)
Supplement: fiac147_Supplemental_Files [file fiac147_supplemental_files.zip › Supp_data_C.docx]

**Supplementary materials**

Figure S1. Species level OTU diversity from Illumina MiSeq metabarcoding data.

Figure S2. Class level diversity from Illumina MiSeq metabarcoding data

Supplmentary data: Extracted values for wild type faecal sequencing results.

| Class |  |  |  |  |  |  |  |  |  |  |  |  |  |  |  |  |  |  |  |
| --- | --- | --- | --- | --- | --- | --- | --- | --- | --- | --- | --- | --- | --- | --- | --- | --- | --- | --- | --- |
| index | Acidobacteria | Actinobacteria | Flavobacteriia | Sphingobacteriia | Bacilli | Alphaproteobacteria | Betaproteobacteria | Gammaproteobacteria | Deinococci | condition |  |  |  |  |  |  |  |  |  |
| 476G1FAECES.A25064.1 | 30 | 135 | 32 | 57 | 48994 | 16 | 33 | 348 | 6 | G1FAECES | |  |  |  |  |  |  |  |  |
|  | 0.060422 | 0.271898 | 0.06445 | 0.114801 | 98.67676 | 0.032225 | 0.066464 | 0.700892 | 0.012084 |  |  |  |  |  |  |  |  |  |  |
|  |  |  |  |  |  |  |  |  |  |  |  |  |  |  |  |  |  |  |  |
| Order |  |  |  |  |  |  |  |  |  |  |  |  |  |  |  |  |  |  |  |
| index | Acidobacteriales | Actinomycetales | Flavobacteriales | Sphingobacteriales | Bacillales | Lactobacillales | Rhizobiales | Burkholderiales | Enterobacteriales | Deinococcales | condition |  |  |  |  |  |  |  |  |
| 476G1FAECES.A25064.1 | 30 | 135 | 32 | 57 | 44 | 48950 | 16 | 33 | 348 | 6 | G1FAECES | |  |  |  |  |  |  |  |
|  | 0.060422 | 0.271898 | 0.06445 | 0.114801 | 0.088619 | 98.58815 | 0.032225 | 0.066464 | 0.700892 | 0.012084 |  |  |  |  |  |  |  |  |  |
|  |  |  |  |  |  |  |  |  |  |  |  |  |  |  |  |  |  |  |  |
| Genus |  |  |  |  |  |  |  |  |  |  |  |  |  |  |  |  |  |  |  |
| index | Unknown Acidobacteriaceae | Actinomyces | Arthrobacter | Nocardioides | Luteimicrobium | Propionibacterium | Myroides | Sphingobacterium | Bacillus | Gracilibacillus | Staphylococcus | Unknown Lactobacillales | Streptococcus | Methylobacterium | Rhizobium | Bordetella | Pseudomonas | Deinococcus | condition |
| 476G1FAECES.A25064.1 | 30 | 7 | 56 | 4 | 53 | 15 | 32 | 57 | 8 | 22 | 14 | 48922 | 28 | 3 | 13 | 33 | 348 | 6 | G1FAECES |
|  |  |  |  |  |  |  |  |  |  |  |  |  |  |  |  |  |  |  |  |
