# Supplementary material for: Gut microbial community supplementation and reduction modulates African armyworm susceptibility to a baculovirus
Source: FEMS Microbiol Ecol. 2022 Dec 6;99(1):fiac147. doi: 10.1093/femsec/fiac147 (PMC9764207; doi:10.1093/femsec/fiac147)
Supplement: fiac147_Supplemental_Files [file fiac147_supplemental_files.zip › Supp_data_Table_S1.docx]

Table S1. Spodoptera exempta nucleopolyhedrovirus (SpexNPV) isolates used in the viral bioassays. Variant type was assigned based on unique RFLP profiles generated from Graham et al (in prep).

| Num | Isolate code | Variant type | Outbreak site | Latitude | Longitude | Date Collected |
| --- | --- | --- | --- | --- | --- | --- |
| 1 | 4MAG2 | 1 | Magole | 6°24' S | 37°23' E | 12/01/2008 |
| 2 | 4MAG5 | 3 | Magole | 6°24' S | 37°23' E | 12/01/2008 |
| 3 | 4MAG11 | 6 | Magole | 6°24' S | 37°23' E | 12/01/2008 |
| 4 | 4MAG16 | 7 | Magole | 6°24' S | 37°23' E | 12/01/2008 |
| 5 | 16EMB1 | 18 | Embukoi | 3°20' S | 37°07' E | 07/04/2008 |
| 6 | 16EMB2 | 12 | Embukoi | 3°20' S | 37°07' E | 07/04/2008 |
| 7 | 16EMB6 | 1 | Embukoi | 3°20' S | 37°07' E | 07/04/2008 |
| 8 | 8KIV2 | 16 | Kivesa | 5°25' S | 38°00' E | 12/02/2008 |
| 9 | 8KIV5 | 16 | Kivesa | 5°25' S | 38°00' E | 12/02/2008 |
| 10 | 8KIV6 | 1 | Kivesa | 5°25' S | 38°00' E | 12/02/2008 |
| 11 | 8KIV9 | 7 | Kivesa | 5°25' S | 38°00' E | 12/02/2008 |
| 12 | 8KIV15 | 11 | Kivesa | 5°25' S | 38°00' E | 12/02/2008 |
| 13 | 9ISH2 | 11 | Mungushi | 3°18' S | 37°08' E | 02/04/2008 |
| 14 | 9ISH3 | 51 | Mungushi | 3°18' S | 37°08' E | 02/04/2008 |
| 15 | 16EMB7 | 18 | Embukoi | 3°20' S | 37°07' E | 07/04/2008 |
| 16 | 16EMB11 | 26 | Embukoi | 3°20' S | 37°07' E | 07/04/2008 |
| 17 | 16EMB16 | 18 | Embukoi | 3°20' S | 37°07' E | 07/04/2008 |
| 18 | 16EMB30 | 33 | Embukoi | 3°20' S | 37°07' E | 07/04/2008 |
| 19 | 16EMB42 | 36 | Embukoi | 3°20' S | 37°07' E | 07/04/2008 |
| 20 | 22LON9 | 54 | Kiva Londo | 3°21' S | 37°09' E | 09/04/2008 |
| 21 | 22LON13 | 33 | Kiva Londo | 5°46' S | 38°11' E | 09/04/2008 |
| 22 | 22LON14 | 12 | Kiva Londo | 5°46' S | 38°11' E | 09/04/2008 |
| 23 | 22LON15 | 53 | Kiva Londo | 5°46' S | 38°11' E | 09/04/2008 |
| 24 | 22LON17 | 32 | Kiva Londo | 5°46' S | 38°11' E | 09/04/2008 |
| 25 | 22LON32 | 54 | Kiva Londo | 5°46'S | 38°11'E | 09/04/2008 |
| 26 | 22LON47 | 57 | Kiva Londo | 3°21' S | 37°09' E | 09/04/2008 |
| 27 | 22LON57 | 18 | Kiva Londo | 5°46' S | 38°11' E | 09/04/2008 |
| 28 | 5BUT13 | 6 | Buti Visaraka | 6°21'S | 37°21' E | 13/01/2008 |
| 29 | 5BUT16 | 11 | Buti Visaraka | 6°21'S | 37°21' E | 13/01/2008 |
| 30 | 5BUT20 | 17 | Buti Visaraka | 6°21' S | 37°21' E | 13/01/2008 |
| 31 | 6MSI1 | 10 | Msimba | 6°44'S | 37°44' E | 14/01/2008 |
| 32 | 6MSI2 | 14 | Msimba | 6°44'S | 37°44' E | 14/01/2008 |
| 33 | 6MSI4 | 11 | Msimba | 6°44' S | 37°44' E | 14/01/2008 |
| 34 | 14MOM2 | 44 | Mombo | 4°54' S | 38°16' E | 27/01/2010 |
| 35 | 14MOM3 | 8 | Mombo | 4°54'S | 38°16' E | 27/01/2010 |
| 36 | 13MAG22 | 6 | Magore | 6°23' S | 37°22' E | 31/12/2009 |
| 37 | 13MAG9 | 13 | Magore | 6°23' S | 37°22' E | 31/12/2009 |
| 38 | 18GEZ19 | 45 | Geza Ulole | 3°20' S | 37°07' E | 08/04/2008 |
| 39 | 18GEZ33 | 49 | Geza Ulole | 3°20' S | 37°07' E | 08/04/2008 |
| 40 | 17LOT30 | 12 | Loto | 4°15' S | 35°29' E | 03/02/2010 |
