# Supplementary material for: Gut microbial community supplementation and reduction modulates African armyworm susceptibility to a baculovirus
Source: FEMS Microbiol Ecol. 2022 Dec 6;99(1):fiac147. doi: 10.1093/femsec/fiac147 (PMC9764207; doi:10.1093/femsec/fiac147)
Supplement: fiac147_Supplemental_Files [file fiac147_supplemental_files.zip › Supp_data_Table_S2.docx]

Table S2. NMDS Results on microbial community composition within each treatment group. NMDS was calculated using Bray-Curtis method, through the *metaMDS* function in R.

|  |  | | | |  | |  | | |  | | |  |
| --- | --- | --- | --- | --- | --- | --- | --- | --- | --- | --- | --- | --- | --- |
|  |  | | NMDS Centroids | | | | | | | |  |  |  |
|  | Factors | | NMDS1 | | | NMDS2 | | NMDS3 | | |  |  |  |
|  | Culture: Probiotic | | -0.1484 | | | -0.0072 | | -0.0544 | | |  |  |  |
|  | Culture: Lab | | 0.4871 | | | 0.0235 | | 0.1784 | | |  |  |  |
|  | Antibiotic: Dosed | | 0.3003 | | | 0.0848 | | 0.1761 | | |  |  |  |
|  | Antibiotic: None | | -0.1041 | | | -0.0294 | | -0.0611 | | |  |  |  |
|  | Viral.Dose: LD80 | | 0.0241 | | | -0.0157 | | -0.0022 | | |  |  |  |
|  | Viral.Dose: None | | -0.1439 | | | 0.0938 | | 0.013 | | |  |  |  |
|  | Dead | | 0.0313 | | | -0.1101 | | 0.0989 | | |  |  |  |
|  | Lived | | -0.0255 | | | 0.0898 | | -0.0806 | | |  |  |  |
|  |  | | | |  | |  | | |  | | |  |
|  | Goodness of fit |  | |  | |  |  | |  |  |  |  |  |
|  |  | r² | | P | |  |  | |  |  |  |  |  |
|  | Culture | 0.114 | | <0.001 | | *** |  | |  |  |  |  |  |
|  | Antibiotic | 0.062 | | <0.001 | | *** |  | |  |  |  |  |  |
|  | Viral.Dose | 0.007 | | 0.327 | |  |  | |  |  |  |  |  |
|  | Dead | 0.026 | | 0.005 | | ** |  | |  |  |  |  |  |
|  |  | | | |  | |  | | |  | | |  |
|  | Number of permutations | | | | 13000 | |  | | |  | | |  |
|  |  | | | |  | |  | | |  | | |  |
